# Low-Cost Avoidance Behaviors are Resistant to Fear Extinction in Humans

**DOI:** 10.3389/fnbeh.2015.00351

**Published:** 2015-12-24

**Authors:** Bram Vervliet, Ellen Indekeu

**Affiliations:** ^1^Department of Psychiatry, Massachusetts General Hospital and Harvard Medical School Boston, MA, USA; ^2^Center for Excellence on Generalization in Health and Psychopathology, University of Leuven—KU Leuven Leuven, Belgium; ^3^Department of Psychology, University of Leuven—KU Leuven Leuven, Belgium

**Keywords:** fear, extinction, avoidance, response prevention, exposure

## Abstract

Elevated levels of fear and avoidance are core symptoms across the anxiety disorders. It has long been known that fear serves to motivate avoidance. Consequently, fear extinction has been the primary focus in pre-clinical anxiety research for decades, under the implicit assumption that removing the motivator of avoidance (fear) would automatically mitigate the avoidance behaviors as well. Although this assumption has intuitive appeal, it has received little scientific scrutiny. The scarce evidence from animal studies is mixed, while the assumption remains untested in humans. The current study applied an avoidance conditioning protocol in humans to investigate the effects of fear extinction on the persistence of low-cost avoidance. Online danger-safety ratings and skin conductance responses documented the dynamics of conditioned fear across avoidance and extinction phases. Anxiety- and avoidance-related questionnaires explored individual differences in rates of avoidance. Participants first learned to click a button during a predictive danger signal, in order to cancel an upcoming aversive electrical shock (avoidance conditioning). Next, fear extinction was induced by presenting the signal in the absence of shocks while button-clicks were prevented (by removing the button in Experiment 1, or by instructing not to click the button in Experiment 2). Most importantly, post-extinction availability of the button caused a significant return of avoidant button-clicks. In addition, trait-anxiety levels correlated positively with rates of avoidance during a predictive safety signal, and with the rate of pre- to post-extinction decrease during this signal. Fear measures gradually decreased during avoidance conditioning, as participants learned that button-clicks effectively canceled the shock. Preventing button-clicks elicited a sharp increase in fear, which subsequently extinguished. Fear remained low during avoidance testing, but danger-safety ratings increased again when button-clicks were subsequently prevented. Together, these results show that low-cost avoidance behaviors can persist following fear extinction and induce increased threat appraisal. On the other hand, fear extinction did reduce augmented rates of unnecessary avoidance during safety in trait-anxious individuals, and instruction-based response prevention was more effective than removal of response cues. More research is needed to characterize the conditions under which fear extinction might mitigate avoidance.

## Introduction

Most studies on fear extinction focus on passive emotional reactions, like freezing in the rat or skin conductance reactivity in humans. Pavlovian fear conditioning first installs these reactions, by pairing a neutral stimulus (the conditioned stimulus, CS) repeatedly with an aversive stimulus (the unconditioned stimulus, US). Over CS-US pairings, the CS starts eliciting de novo fear reactions, in anticipation of the US. Once these reactions are firmly established, they can be removed again by repeatedly presenting the CS in the absence of its US, which leads to a gradual decline of the CS-elicited fear reactions (i.e., fear extinction). Pavlovian fear conditioning and extinction serve as widely used translational models to study the psychobiological mechanisms of the development and treatment of clinical anxiety (Milad and Quirk, 2012). According to the Pavlovian conditioning model, irrational fears stem from erroneous associations between intrinsically safe situations (CS) and imagined dangerous consequences (US). Therefore, exposure-based treatments use the fear extinction principle to counter these erroneous associations and decrease the irrational fears, by exposing the patient to the objects/situations of fear over and over again (Vervliet et al., 2013). Meta-analyses of clinical studies have confirmed the overall efficacy of this technique for reducing fear, although relatively high rates of both non-responding and relapse pose continuous, serious challenges (Craske and Mystkowski, 2006).

Anxiety disorders are characterized by elevated fears of safe situations, as well as excessive avoidance of those situations (American Psychiatric Association, 2013). Avoidance is a form of self-protective action that serves to minimize confrontations with a feared danger (for a review, see Krypotos et al., 2015). Although avoidance is often adaptive in the face of real danger, it is superfluous when the fears are irrational and the danger absent. Moreover, it maintains these irrational fears by precluding learning opportunities that could otherwise show the actual absence of danger and produce fear extinction (Lovibond et al., 2009; see also Krypotos et al., 2015). Persistent avoidance is therefore not only a cardinal symptom across the anxiety disorders, but also a major reason why irrational fears do not extinguish spontaneously in the anxiety patient. An important part of exposure-based treatments is to identify and neutralize avoidance behaviors prior to conducting exposures to the feared situations, in order to optimize the extinction learning process (termed “response prevention with extinction,” RPE). The success of exposure-based treatment is determined by reductions in fear as well as avoidance. Some even claim that reducing avoidance is the only relevant outcome measure of anxiety treatments (Hayes et al., 2006). In contrast, contemporary fear extinction research focuses almost exclusively on removing passive fear reactions, with no inclusion of avoidance in the fear conditioning history or during the extinction test phase. Hence, little is known about the effects of fear extinction on avoidance extinction. It remains unclear, e.g., to what extent behavioral and/or pharmacological enhancers of fear extinction might also mitigate avoidance.

Mitigating rates of avoidance was a major focus of pre-clinical animal research in the 1960s–1970s, and RPE was the most investigated treatment at the time (also termed “flooding”). In a seminal study on avoidance learning with high intense shocks in dogs, Solomon et al. (1953) found that preventing the avoidance response (jumping over a hurdle avoided the shock, a glass-barrier prevented the jumping) led to avoidance extinction only in 7 out of 9 dogs when the glass-barrier was removed. Later studies with less intense shocks and rats as subjects showed that RPE does speed up later avoidance extinction compared to rats that received extinction without response prevention or no treatment (reviewed by Mineka, 1979). Unfortunately, these early studies only reported the number of trials-to-criterion of extinction, but did not report initial rates of avoidance responding during test. Nevertheless, the fact that avoidance extinction was never immediate suggests that the avoidance response initially returned when the prevention was lifted, before entering into extinction. This was recently confirmed by a behavioral conflict study in which hungry rats had to chose between pressing a lever for food under threat of shock vs. jumping on a platform that protected against the shock but with no food available (Bravo-Rivera et al., 2015). The safe platform constituted a costly avoidance response as it implied the loss of food (high-cost avoidance). Removal of the platform (and the shocks) initially increased fear-related freezing that subsequently extinguished (response prevention with extinction). Despite complete fear extinction, returning the platform to the cage triggered significant return of shock-avoidance responses (even in the absence of actual shocks). Moreover, the amount of return correlated with c-Fos measured neural activity in the prelimbic prefrontal cortex and the ventral striatum (brain regions closely linked to anxiety and avoidance), but not in the infralimbic prefrontal cortex or the basolateral amygdala (brain regions closely linked to fear extinction). These results show that avoidance behaviors in the rat can persist irrespective of fear extinction.

A seminal study on shock-avoidance conditioning in humans confirmed that preventing an established avoidance response triggers a return of conditioned fear responses (Lovibond et al., 2009), but subsequent fear extinction and its effect on avoidance were not examined. To date, this issue remains untested in humans, despite its clinical relevance. For that purpose, we merged components of the avoidance protocol of Lovibond et al. (2009) with components of a widely used fear extinction protocol (Milad et al., 2005). Of note, the (Lovibond et al., 2009) protocol involves a low-cost avoidance response (merely clicking a button with no associated costs), which differs from the costly avoidance response in the Bravo-Rivera et al. (2015) study. Arguably, clinical avoidance comprises both high- and low-cost avoidance behaviors that prevent extinction and maintain anxiety in the long run. Subtle safety behaviors like carrying anxiety pills are an example of low-cost avoidance that can go unnoticed and are sometimes difficult to treat. Moreover, because of the low cost, these avoidance behaviors may be especially persistent and unaffected by fear extinction. This, in turn, may pose a continuous vulnerability for relapse of the fear and avoidance symptomatology. For these reasons, we focused on low-cost avoidance to investigate the effects of response prevention and extinction.

Two different colorings of a lamp in a room picture signaled the imminence of an aversive electrical shock, or nothing. Skin conductance responses and danger-safety ratings tracked the development of anticipatory arousal and threat appraisal to the lamp colorings. Mouse-clicking a button on the room pictures served to avert the shock. We operationalized response prevention by removing the button (Experiment 1) or by instructing participants that the button was no longer available (Experiment 2), while shocks were no longer delivered (extinction). We counterbalanced two different lenghts of extinction in each experiment, in order to minimize the chances that spontaneous fluctuations of fear would contribute to persistent avoidance. For the critical test of this study, we assessed persistent avoidance by re-introducing the (availability of the) avoidance button and by recording the number of button-clicks accordingly. Based on the Bravo-Rivera et al. findings, we predicted a return of avoidance following fear extinction. For exploratory purposes, we next removed the (availability of the) avoidance button again to test the persistence of fear extinction. Finally, we explored relationships between anxiety- and avoidance-related personnality traits (measured by validated questionnaires) and rates of avoidance responses before and after fear extinction (cf. Lommen et al., 2010; van Meurs et al., 2014).

## Experiment 1

## Materials and methods

### Participants

Twenty individuals (age 17–21, average = 18.9, 18 females), mostly from first grade psychology, participated to earn course credis or financial compensation (8 EUR). Given the administration of electrocuaneous shocks in the experiment, participants were screened and excluded for the following conditions: pregnancy, cardivascular, pneuomological, neurological or other serious medical conditions, psychiatric conditions, chronic pain near the wrists, electronic implants, or having received medical instructions to avoid stressful situations. Participants were randomly assigned to Group Long-Ext and Group Short-Ext. The study was approved by the Social and Societal Ethical Committee and the Medical Ethical Committee of the University of Leuven–KU Leuven. All participants gave informed consent and were informed that they could decline further participation at any time during the experiment.

### Materials

The conditional stimuli were pictures of an office room with an desk top lamp that could color yellow or blue (taken from Milad et al., 2005), presented on a computer screen located on eye-level in front of the participant at approximately 500 mm. The avoidance stimulus was a picture of a red button that could appear over the room pictures (top left). Danger-safety ratings were measured on a trial-by-trial basis during each room picture presentation. A vertical scale was presented on the left of the screen with three options from low to high: “Safe, Uncertain, Danger” (translated from Dutch). Participants could move over the scale by using the computer mouse, and completed their rating by clicking on the left mouse button. A 2 ms electrocutaneous shock delivered to the forearm of the left hand served as unconditional stimulus (US). It was administered by a Digitimer DS7A constant current stimulator (Hertfordshire, UK) via a pair of 11-mm Fukuda Standard AG/AGCl electrodes, filled with K-Y Jelly. The intensity of the shock was individually selected to a level where it was “uncomfortable but not painful.” Participants were seated in an armchair in a sound attenuated room, adjacent to the experimenter's room.

Electrodermal activity was recorded using a skin conductance coupler manufactured by Colbourn Instruments (model V71-23, Allentown, PA). During skin conductance measurement, the coupler applied a constant voltage of 0.5 V across a pair of sintered-pellet silver chloride electrodes (8 mm), attached to the hypothenar palm of the left hand. The inter-electrode distance was approximately 10 mm. The electrodes were filled with K-Y Jelly. The resulting conductance signal was submitted through a Labmaster DMA 12-bit analog-to-digital converter (Scientific Solutions, Solon, Ohio) and digitized at 10 Hz from 2 s prior to CS onset until 6 s after CS offset.

#### Trait portion of the state-trait anxiety inventory (STAI-T)

The STAI measures trait anxiety (STAI-T) via 20 questions with scores ranging from 20 to 80, with higher scores indicating higher levels of anxiety (Spielberger et al., 1970). The Dutch version by van der Ploeg (2000) was used, which has good reliability and validity.

#### Cognitive-behavioral avoidance scale (CBAS)

This 31 item questionnaire measures four dimensions of avoidance: Cognitive-Social, Cognitive-Nonsocial, Behavioral-Social, and Behavioral-Nonsocial (Ottenbreit and Dobson, 2004). The total CBAS score correlates highly with depression and anxiety inventories (e.g., STAI). The Dutch version by Vandromme et al. (2007) was used, which shows good reliability and validity.

#### Intolerance of uncertainty scale (IU)

This 27 time questionnaire measures emotional, cognitive and behavioral reactions to ambiguous situations, implications of being uncertain, and attempts to control the future (Freeston et al., 1994). The Dutch version by de Bruin et al. (2006) was used, which shows good reliability and validity.

### Procedure

Following general instructions (about the use of pictures and electrical shocks in the experiment, and the measurement of skin conductance) and completion of the informed consent, participants were fitted with electrodes and were led through the work-up procedure to select a “definitely uncomfort- able, but not painful” shock level. Next, participants received explicit instructions that the blue lamp would signal the electrical shock, and that the yellow would signal the absence of the electrical shock (this was done to ensure a fast development of fear reactions to the CSP with a minimal number of actual CS-US conditioning trials). The danger-safety ratings scale and the red button were explained to them (move the pointer over the desired location and mouse-click).

**Figure 1:**
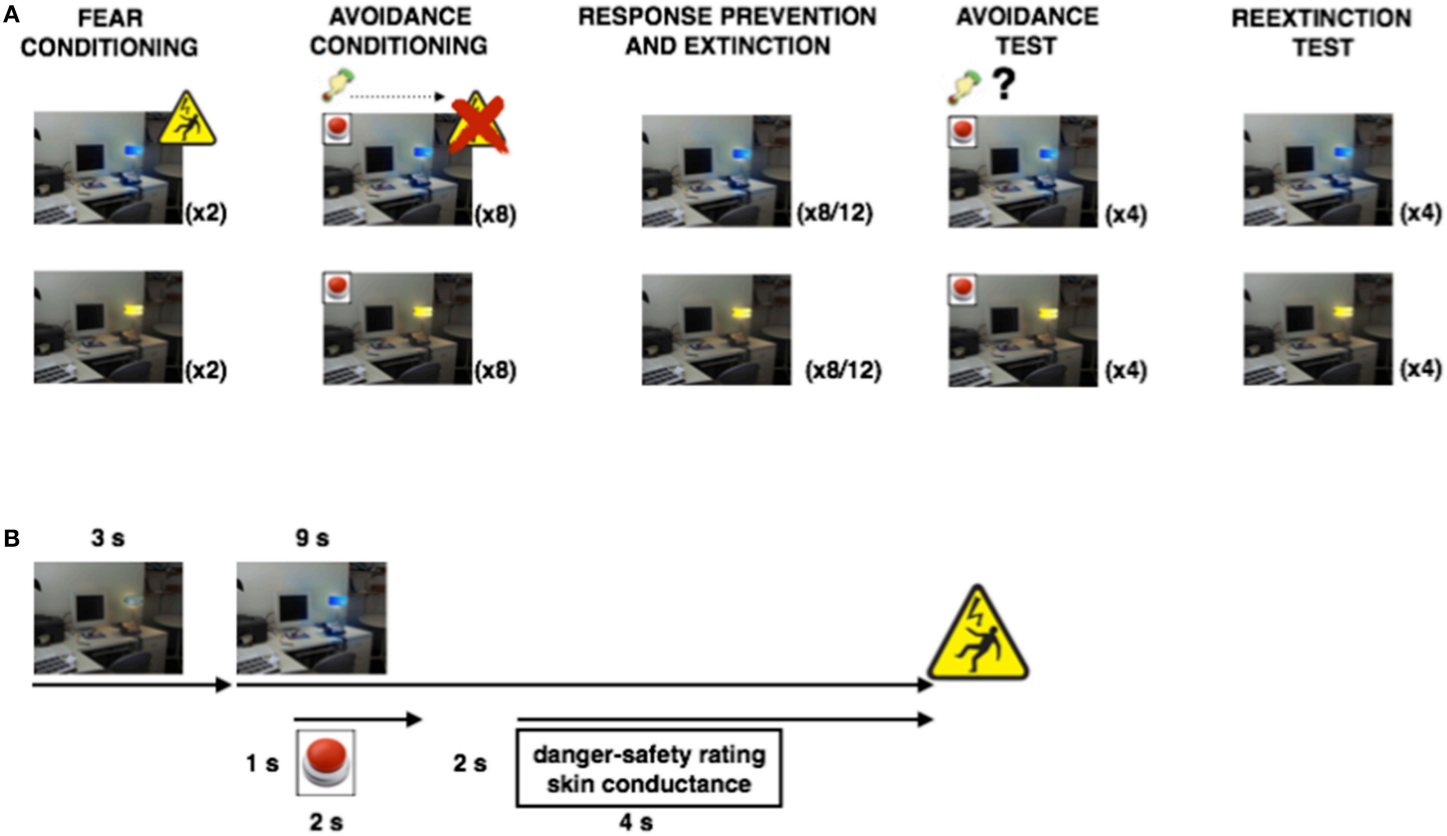
**Design of Experiment 1**. **(A)**: Overview of the experimental phases. During Fear Conditioning, blue colorings of the desktop lamp were followed by the aversive shock, while yellow colorings were not (two trials each). During Avoidance Conditioning, clicking the newly added button canceled the shock to blue lamp colorings (eight trials each). Both the button and the shocks were subsequently removed in the Response Prevention and Extinction phase (8 or twelve trials each). In order to assess the persistence of avoidance responding, the button (but not the shock) reappeared during both colors in the Avoidance Test phase (four trials each). Removing the button again during both colors in the final Reextinction Test phase probed the persistence of fear extinction following renewed avoidance availability (four trials each). **(B)**: Timeline of an avoidance conditioning trial. The room picture (context) is presented for 3 s, before the desktop lamp colors blue (or yellow) for 9 s. One second after lamp coloring, a red button appears for 2 s (during which the participant can choose to click the button using the computer mouse). Two seconds after removal of the button, a mouse-controlled vertical rating scale appears on the left of the screen comprising three levels of increasing threat: Safe (lowest)—Uncertain (middle)—Danger (highest). Finally, the aversive shock is administered at picture offset (unless the participant clicked the button earlier).

The room pictures were always presented for 12 s. Three seconds after room picture onset, the lamp colored yellow or blue for the remaining 9 s. One second after lamp coloring onset, the red button appeared for 2 s (on trials that contained the red button). Two seconds later, the rating scale appeared until the participant clicked on it or until the picture disappeared from the screen (during Pavlovian conditioning, the rating scale appeared 2 s earlier). The electrical shock was delivered at 500 ms before picture offset (on CSP trials during some phases). Inter-trial intervals varied between 13 and 17 s, with a mean of 15 s. The experiment consisted of five phases: Fear conditioning, avoidance conditioning, fear extinction with response prevention, avoidance test, and reextinction test (see Figure 1). All phases occurred consecutively without interruptions. The fear conditioning phase consisted of two presentations of the yellow and the blue light, where the blue light (CSP) was always followed by the US, while the yellow light was not (CSM). During the subsequent avoidance conditioning phase, the red button appeared during all eight CSP and CSM presentations. Next, both the button and the USs were removed during the CSP and CSM presentations of the fear extinction and response prevention phase (eight presentations of each CS in group Extinction Short and 12 presentations of each CS in group Extinction Long). In order to measure the recovery of avoidance behavior, the red button returned during the four CSP and CSM presentations of the avoidance test phase (no shock administrations). In order to measure residual skin conductance and shock-expectancy, the button was removed again during the four CSP and CSM presentations of the reextinction test phase. The numbers of trials for each phase were chosen based on standard fear conditioning, extinction and avoidance studies that include consideration of requirements for skin conductance measurements (e.g., Lovibond et al., 2013; Vervliet and Geens, 2014).

## Results

Skin conductance responses (SCRs) were calculated by subtracting trial-by-trial baseline levels (average skin conductance level during 2 s window prior to context presentation) from peak levels (maximal skin conductance level during 4 s window prior to CS offset). Negative responses were replaced by zero (cf. Soeter and Kindt, 2010). Prior to statistical analyses, SCRs were Z-transformed per participant across all phases. Avoidance reponses were scored as 1 (vs. 0) and averaged per participant, per CS and per phase prior to statistical analyses. The vertical rating scale comprised three categories denoting increasing threat value: safe (lowest)—uncertain (middle)—danger (highest), which we considered to be an interval scale allowing parametric testing (analysis of variance, ANOVA). Nevertheless, to account for the possibility that the scale may only be ordinal, we additionally performed non-parametric tests (Wilcoxon signed rank test) for crucial comparisons (effects of adding/removing the button).

### Avoidance responses

**Figure 2:**
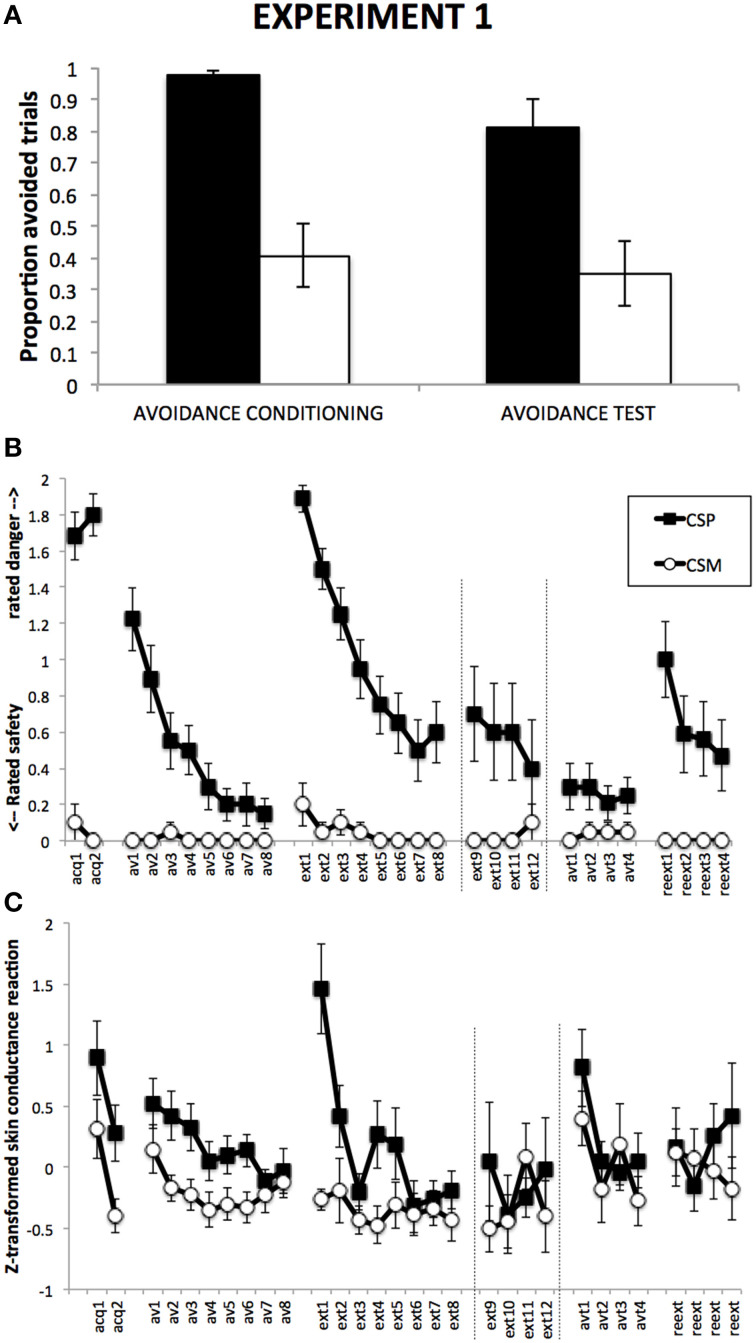
**Results from Experiment 1**. **(A)**: Proportions of CSP and CSM trials during which the avoidance button was clicked, for avoidance conditioning and avoidance test phase separately. **(B)**: Mean shock-expectancy ratings during CSP and CSM (0 = “safe,” 1 = “uncertain,” 2 = “danger”), for all trials of Pavlovian conditioning (acq1-2), avoidance conditioning (av1-8), response prevention and extinction (ext1-8) with extension for Group ExtLong between the dashed lines (ext9-12), avoidance test (avt1-4), and reextinction (reext1-4). **(C)**: Z-transformed skin conductance reactions during CSP and CSM during all trials (cf. **B**). Error bars represent standard errors of the means.

Figure 2A suggests a higher proportion of avoided CSP trials compared to CSM trials, which remains during Avoidance test, despite a general decrease. This was confirmed by a 2 (Group) × 2 (CS) × 2 (Phase) RM-ANOVA, revealing a main effect of CS, *F*_(1, 18)_ = 33.04, *p* < 0.001, η^2^ = 0.65, a main effect of Phase, *F*_(1, 18)_ = 4.60, *p* < 0.05, with no CS × Phase interaction, *F*_(1, 18)_ = 1.01, *p* = 0.33.

### Danger-safety ratings

#### Fear conditioning

Figure 2B suggests robust differential danger-safety ratings with no difference between the groups, which was confirmed by a 2 (Group) × 2 (CS) × 2 (Trial) RM-ANOVA, revealing a main effect of CS, *F*_(1, 17)_ = 104.46, *p* < 0.001, η^2^ = 0.86, with no CS × Group interaction, *F*_(1, 17)_ = 1.94, *p* = 0.18.

#### Avoidance conditioning

Figure 2B suggests gradual decrease of danger-safety ratings over avoidance trials, which was confirmed by a 2 (Group) × 2 (CS) × 8 (Trial) RM-ANOVA, revealing a main effect of CS, *F*_(1, 14)_ = 25.17, *p* < 0.001, η^2^ = 0.64, and a significant CS × Trial interaction, *F*_(7, 98)_ = 8.48, *p* < 0.001, η^2^ = 0.38, with significant linear and quadratic trends, *F*_(1, 14)_ = 22.24, *p* < 0.001, η^2^ = 0.61, and *F*_(1, 14)_ = 8.53, *p* < 0.05, η^2^ = 0.38, respectively. The Group × CS × Trial interaction was not significant, *F*_(7, 98)_ = 1.69, *p* = 0.12, suggesting similar decrease of danger-safety rating across the two groups.

#### Transition from avoidance conditioning to response prevention

Figure 2B suggests a strong return of differential danger-safety ratings upon removal of the avoidance button, which was confirmed by a 2 (Group) × 2 (CS) × 2 (Trial: last avoidance trial, first extinction trial) RM-ANOVA that revealed a CS × Trial interaction, *F*_(1, 16)_ = 79.34, *p* < 0.001, η^2^ = 0.83, which was unexpectedly qualified by a marginally significant Group × CS × Trial interaction, *F*_(1, 16)_ = 3.82, *p* = 0.07, η^2^ = 0.19. A 2 (Group) × 2 (CS) × 2 (Trial: last Pavlovian, first extinction) RM-ANOVA further revealed that the level of differential danger-safety ratings was statistically indistinguishable from the Pavlovian conditioning phase, as suggested by the absence of a CS × Trial interaction, *F*_(1, 16)_ = 0.44, *p* = 0.52. Again, the Group × CS × Trial was unexpectedly signficant, *F*_(1, 16)_ = 8.17, *p* < 0.05, η^2^ = 0.34, suggesting stronger return in Group LongExt vs. Group ShortExt although the two groups had received identical treatment up to this point. A Wilcoxon signed rank test comparing the last CSP avoidance trial with the first CSP extinction trial confirmed that ratings shifted to higher scale-categories upon removal of the avoidance button, Z = −3.88, *p* < 0.001.

#### Response prevention and extinction

Focusing on the first eight trials of extinction, Figure 2B suggests gradual decrease of danger-safety ratings. This was confirmed by a 2 (Group) × 2 (CS) × 8 (Trial) RM-ANOVA, revealing a main effect of CS, *F*_(1, 15)_ = 147.67, *p* < 0.001, η^2^ = 0.91, and a significant CS × Trial interaction, *F*_(7, 105)_ = 7.68, *p* < 0.001, η^2^ = 0.34. Unexpectedly, the Group × CS interaction was also significant, *F*_(1, 15)_ = 14.16, *p* < 0.01, η^2^ = 0.49, suggesting more differential danger-safety ratings in Group LongExt (see Figure 1), but the Group × CS × Trial interaction was not significant, *F*_(7, 105)_ = 0.45, *p* = 0.87, confirming similar extinction curves across the two groups. In order to compare the end-points of extinction between the groups, we compared the last 4 extinction trials in a 2 (Group) × 2 (CS) × 4 (Trial: ext5-8 in Group ShortExt, ext9-12 in Group LongExt), revealing a main effect of CS, *F*_(1, 18)_ = 10.00, *p* < 0.01, η^2^ = 0.36, with no Group × CS interaction, *F*_(1, 18)_ = 0.25, *p* = 0.62. This suggests that extinction was not complete, equally so in both groups.

#### Avoidance test

Figure 2B suggests that the return of the avoidance button had no detectable impact on the level of differential danger-safety ratings. We calculated the average danger-safety ratings for each CS during the four avoidance test trials and compared this with the averaged last four extinction trials (ext5-8 in Group ShortExt, ext9-12 in Group LongExt). The resulting 2 (Group) × 2 (CS) × 2 (Phase: last extinction trials vs. avoidance test trials) revealed a main effect of CS, *F*_(1, 18)_ = 11.51, *p* < 0.01, η^2^ = 0.39, and a marginally significant CS × Phase interaction, *F*_(1, 18)_ = 4.35, *p* = 0.052, η^2^ = 0.20, suggesting a further decrease in differential danger-safety ratings when the avoidance operant was made available again.

#### Reextinction test

Figure 2B suggests an increase of differential danger-safety ratings upon removal of the avoidance button, which was confirmed by a 2 (Group) × 2 (CS) × 2 (Trial: last avoidance test trial, first reextinction test trial) RM-ANOVA, revealing a significant main effect of CS, *F*_(1, 15)_ = 23.77, *p* < 0.001, η^2^ = 0.61, and most importantly, a significant CS × Trial interaction, *F*_(1, 15)_ = 16.64, *p* < 0.01, η^2^ = 0.53, that was similar across the two groups, Group × CS × Trial, *F*_(1, 15)_ = 0.06, *p* = 0.81. Moreover, differential danger-safety ratings also increased against the last extinction trial prior to avoidance test, as confirmed by a 2 (Group) × 2 (CS) × 2 (Trial: last extinction and first post-avoidance test) RM-ANOVA, which revealed a main effect of CS, *F*_(1, 15)_ = 14.54, *p* < 0.01, η^2^ = 0.49, a main effect of Trial, *F*_(1, 15)_ = 9.93, *p* < 0.01, η^2^ = 0.40, and most importantly, a significant CS × Trial interaction, *F*_(1, 15)_ = 8.53, *p* < 0.05, η^2^ = 0.36, that was not qualified by Group, *F*_(1, 15)_ = 1.71, *p* = 0.21. Post hoc comparisons revealed that the CS × Trial interaction was driven by a significant increase of danger-safety ratings to the CSP, *F*_(1, 15)_ = 9.94, *p* < 0.01, η^2^ = 0.40, while danger-safety ratings to CSM did not change, *F*_(1, 15)_ = 1.13, *p* = 0.30. A Wilcoxon signed rank test comparing the last CSP extinction trial with the first CSP reextinction trial confirmed that ratings shifted to higher scale-categories following the avoidance test, *Z* = −2.46, *p* < 0.05.

### Skin conductance

#### Fear conditioning

Figure 2C suggests successful conditioning of differential SCR in both groups, which is confirmed by a 2 (Group) × 2 (CS, averaged over the two trials) RM-ANOVA, revealing a main effect of CS, *F*_(1, 18)_ = 7.74, *p* = 0.01, η^2^ = 0.30.

#### Avoidance conditioning

Figure 2C suggests differential SCR with a general decrease over trials, which is confirmed by a 2 (Group) × 2 (CS) × 8 (Trial) RM-ANOVA, revealing a main effect of CS, *F*_(1, 18)_ = 12.12, *p* < 0.01, η^2^ = 0.40, and a main effect of Trial, *F*_(7, 126)_ = 2.59, *p* < 0.05, η^2^ = 0.13, with no CS × Trial interaction, *F*_(7, 126)_ = 0.79, *p* = 0.59.

#### Transition from avoidance conditioning to response prevention

Figure 2C suggests an increase of differential SCR upon removal of the avoidance button, which is confirmed by a 2 (Group) × 2 (CS) × 2 (Trial: last avoidance trial, first extinction trial) RM-ANOVA, revealing a main effect of CS, *F*_(1, 18)_ = 17.92, *p* < 0.01, η^2^ = 0.50, a main effect of Trial, *F*_(1, 18)_ = 12.30, *p* < 0.01, η^2^ = 0.41, and most importantly, a significant CS × Trial interaction, *F*_(1, 18)_ = 11.59, *p* < 0.01, η^2^ = 0.39.

#### Response prevention and extinction

Focusing on the first eight trials, Figure 2C suggests a gradual extinction of differential SCR, which is confirmed by a 2 (Group) × 2 (CS) × 8 (Trial) RM-ANOVA, revealing a main effect of CS, *F*_(1, 18)_ = 38.94, *p* < 0.001, η^2^ = 0.68, a main effect of Trial, *F*_(7, 126)_ = 4.31, *p* < 0.01, η^2^ = 0.19, and most importantly a significant CS × Trial interaction, *F*_(7, 126)_ = 3.46, *p* < 0.01, η^2^ = 0.16. In order to compare the end-points of extinction between the groups, we compared the last 4 extinction trials in a 2 (Group) × 2 (CS) × 4 (Trial: ext5-8 in Group ShortExt, ext9-12 in Group LongExt), revealing a main effect of CS, *F*_(1, 18)_ = 4.93, *p* < 0.05, η^2^ = 0.22, with no Group × CS interaction, *F*_(1, 18)_ = 0.85, *p* = 0.37. This suggests that extinction was not complete, equally so in both groups.

#### Avoidance test

Figure 2C suggests that the return of the avoidance button had no detectable impact on the level of differential SCR, but produced a general decrease of SCR. We calculated the average SCR for each CS during the four avoidance test trials and compared this with the averaged last four extinction trials (ext5-8 in Group ShortExt, ext9-12 in Group LongExt). The resulting 2 (Group) × 2 (CS) × 2 (Phase: last extinction trials vs. avoidance test trials) revealed a marginally significant main effect of CS, *F*_(1, 18)_ = 3.43, *p* = 0.08, η^2^ = 0.16, a significant main effect of Phase, *F*_(1, 18)_ = 12.78, *p* < 0.01, η^2^ = 0.42, but no CS × Phase interaction, *F*_(1, 18)_ = 0.22, *p* = 0.64, suggesting an overall decrease in SCR.

#### Reextinction test

Figure 2C suggests no return of differential SCR, but a further decrease in overall SCR. This was confirmed by a 2 (Group) × 2 (CS) × 2 (Trial: last extinction trial vs. first reextinction test trial), revealing only a significant effect of Trial, *F*_(1, 16)_ = 6.10, *p* < 0.05, η^2^ = 0.28, and by a 2 (Group) × 2 (CS) × 2 (Phase: mean last four extinction trials vs. mean post-avoidance test) RM-ANOVA, revealing only a significant main effect of Trial, *F*_(1, 16)_ = 9.25, *p* < 0.01, η^2^ = 0.37. Both the 2 (Group) × 2 (CS) × 2 (Trial: last avoidance test vs. first post-avoidance test) and the 2 (Group) × 2 (CS) × 2 (Phase: avoidance test vs. post-avoidance test) RM-ANOVAs revealed no significant effects.

## Discussion

Experiment 1 was set up to validate a response prevention and extinction (RPE) protocol in human avoidance conditioning, and to assess the effects of a return of avoidance availability on avoidance frequency and conditioned fear responding. Following differential fear conditioning (CSP/CSM), participants learned to produce the avoidance response primarily to the danger cue CSP and less so to the safety cue CSM. Shock-expectancy and SCR gradually decreased over avoidance trials, but sudden removal of the avoidance availability (response prevention) elicited a strong return of shock-expectancy and SCR that gradually decreased over the extinction trials. These results are in line with typical observations in exposure treatment (initial increase of anxiety, followed by extinction) as well as in animal RPE research. Moreover, the results revealed that a return of avoidance availability triggered a return of avoidance responding to the danger cue CSP. Subsequent removal of the avoidance availability produced an increase in shock-expectancy relatively to the end of RPE. Together, these results suggest that RPE effects are difficult to generalize to the original situation without response prevention, as evidenced by a return of avoidance and shock-expectancy.

## Experiment 2

The addition/removal of the avoidance button constituted a salient visual event in Experiment 1 that may have hindered generalization of RPE effects across the different phases (Nakajima, 2014). During exposure treatment, on the other hand, response prevention is often accomplished by *instructing* patients not to engage in avoidance activities, rather than physically removing their availability altogether (e.g., instructing to sit far away from the exit during agoraphobic exposure exercises in a theater). Hence, we decided to use verbal instructions to indicate the (un)availability of the avoidance button in Experiment 2, while the button featured during all phases of the experiment (except for the initial Pavlovian conditioning phase). Otherwise, Experiment 2 was exactly identical to Experiment 1.

## Materials and methods

### Participants

Twenty individuals (age 18–23, average age = 19.2, 14 females) participated in the experiment. Enrollment, screening and exclusion criteria were exactly identical as Experiment 1.

### Apparatus

Identical to Experiment 1.

### Procedure

The procedure was identical to Experiment 1, except for the fact that the red button was also present during the Extinction with Response Prevention phase and the Reextinction Test phase, while these phases were preceded by written instructions on the screen: “Please don't click the red button from now on.” Participants maintained control over the mouse and could, in principle, still click the button. The Avoidance Test phase was preceded by the following instructions: “You are free to click the red button from now on.”

## Results

### Avoidance responses

**Figure 3:**
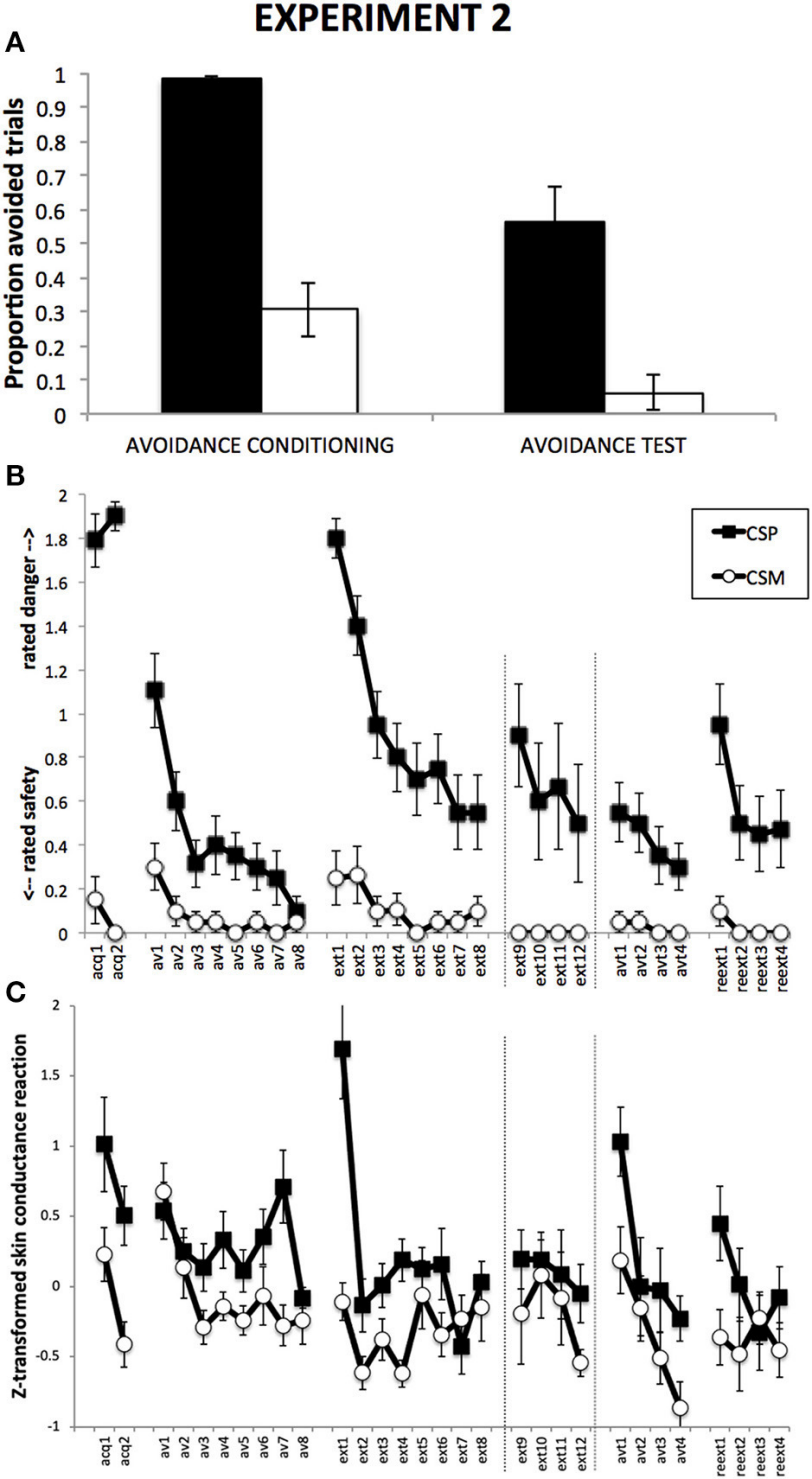
**Results from Experiment 2**. **(A)**: Proportions of CSP and CSM trials during which the avoidance button was clicked, for avoidance conditioning and avoidance test phase separately. **(B)**: Mean shock-expectancy ratings during CSP and CSM (0 = “safe,” 1 = “uncertain,” 2 = “danger”), for all trials of Pavlovian conditioning (acq1-2), avoidance conditioning (av1-8), response prevention and extinction (ext1-8) with extension for Group ExtLong between the dashed lines (ext9-12), avoidance test (avt1-4), and reextinction (reext1-4). **(C)**: Z-transformed skin conductance reactions during CSP and CSM during all trials (cf. **B**). Error bars represent standard errors of the means.

Figure 3A suggests a higher proportion of avoided CSP trials compared to CSM trials, which remains during Avoidance test despite a general decrease in responding. This was confirmed by a 2 (Group) × 2 (CS) × 2 (Phase) RM-ANOVA, revealing a main effect of CS, *F*_(1, 18)_ = 68.03, *p* < 0.001, η^2^ = 0.79, a main effect of Phase, *F*_(1, 18)_ = 24.31, *p* < 0.001, with no CS × Phase interaction, *F*_(1, 18)_ = 2.04, *p* = 0.17. The data show the absence of button-clicks during the Response Prevention and Extionction Phase or during the Reextinction Test phase.

### Danger-safety ratings

#### Fear conditioning

Figure 3B suggests robust differential danger-safety ratings, which was confirmed by a 2 (ShortExt, LongExt) × 2 (CS+, CS−) × 2 (Trials) repeated measures ANOVA, revealing a main effect of CS, *F*_(1, 17)_ = 262.01, *p* < 0.001, η^2^ = 0.94, with no interaction with Group, *F*_(1, 17)_ = 1.77, *p* = 0.20.

#### Avoidance conditioning

Figure 3B suggests gradual decrease of differential danger-safety ratings over avoidance conditioning trials. This was confirmed by a 2 (Group) × 2 (CS) × 8 (Trial) RM-ANOVA, revealing a significant main effect of CS, *F*_(1, 16)_ = 13.97, *p* < 0.01, η^2^ = 0.47, as well as a significant CS × Trial interaction, *F*_(7, 112)_ = 3.55, *p* < 0.01, η^2^ = 0.18. Unexpectedly, this interaction was qualified by Group, *F*_(7, 112)_ = 2.19, *p* < 0.05, η^2^ = 0.12.

#### Transition from avoidance conditioning to response prevention

Figure 3B suggests a strong return of differential danger-safety ratings upon removal of the avoidance button, which was confirmed by a 2 (Group) × 2 (CS) × 2 (Trial: last avoidance trial, first extinction trial) RM-ANOVA that revealed a CS × Trial interaction, *F*_(1, 18)_ = 119.12, *p* < 0.001, η^2^ = 0.87, with no Group × CS × Trial interaction, *F*_(1, 18)_ = 0.53, *p* = 0.47. A 2 (Group) × 2 (CS) × 2 (Trial: last fear conditioning trial, first extinction trial) RM-ANOVA further revealed that this return of differential danger-safety ratings was not complete, evidenced by the signficant CS × Trial interaction, *F*_(1, 18)_ = 6.79, *p* = 0.05, with no Group × CS × Trial interaction, *F*_(1, 18)_ = 0.14, *p* = 0.71. A Wilcoxon signed rank test comparing the last CSP avoidance trial with the first CSP extinction trial confirmed that ratings shifted to higher scale-categories upon removal of the avoidance button, *Z* = −4.10, *p* < 0.001.

#### Response prevention and extinction

Focusing on the first eight trials of extinction, Figure 3B suggests gradual decrease of danger-safety ratings. This was confirmed by a 2 (Group) × 2 (CS) × 8 (Trial) RM-ANOVA, revealing a main effect of CS, *F*_(1, 16)_ = 36.57, *p* < 0.001, η^2^ = 0.70, and a significant CS × Trial interaction, *F*_(7, 112)_ = 11.93, *p* < 0.001, η^2^ = 0.43, with no Group × CS × Trial interaction, *F*_(7, 112)_ = 0.23, *p* = 0.98. In order to compare the end-points of extinction between the groups, we compared the last four extinction trials in a 2 (Group) × 2 (CS) × 4 (Trial: ext5-8 in Group ShortExt, ext9-12 in Group LongExt), revealing a significant main effect of CS, *F*_(1, 17)_ = 12.01, *p* < 0.01, η^2^ = 0.41, with no Group interaction, *F*_(1, 17)_ = 0.25, *p* = 0.63. This suggest incomplete extinction, equally so across the two groups.

#### Avoidance test

Figure 3B suggests that the return of the avoidance button had no detectable impact on the level of differential danger-safety ratings. We calculated the average danger-safety ratings for each CS during the four avoidance test trials and compared this with the averaged last four extinction trials (ext5-8 in Group ShortExt, ext9-12 in Group LongExt). The resulting 2 (Group) × 2 (CS) × 2 (Phase: last extinction trials vs. avoidance test trials) revealed a significant main effect of CS, *F*_(1, 18)_ = 22.05, *p* < 0.001, η^2^ = 0.55, with no CS × Phase interaction, *F*_(1, 18)_ = 1.18, *p* = 0.29.

#### Reextinction test

Figure 3B suggests an increase of differential danger-safety ratings upon removal of the avoidance button, which was confirmed by a 2 (Group) × 2 (CS) × 2 (Trial: last avoidance test, first reextinction test) RM-ANOVA, revealing a significant main effect of CS, *F*_(1, 17)_ = 22.74, *p* < 0.001, η^2^ = 0.57, and most importantly, a significant CS × Trial interaction, *F*_(1, 17)_ = 15.26, *p* < 0.001, η^2^ = 0.47, that was qualified by Group, *F*_(1, 17)_ = 6.78, *p* = 0.02, η^2^ = 0.29. Separate CS × Trial RM-ANOVAs per group confirmed a significant return of differential danger-safety ratings in Group ShortExt, *F*_(1, 8)_ = 12.00, *p* < 0.01, η^2^ = 0.60, but not in Group LongExt, *F*_(1, 9)_ = 2.25, *p* = 0.17. Moreover, differential danger-safety ratings also increased against the last extinction trial prior to avoidance test, as evidence by a 2 (Group) × 2 (CS) × 2 (Trial: last extinction, first post avoidance test) RM-ANOVA, revealing a main effect of CS, *F*_(1, 18)_ = 17.04, *p* < 0.01, η^2^ = 0.49, and a CS × Trial interaction, *F*_(1, 18)_ = 5.19, *p* < 0.05, η^2^ = 0.22, which was not qualified by Group, *F*_(1, 18)_ = 0.11, *p* = 0.75. *Post-hoc* comparisons revealed that the CS × Trial interaction was driven by a significant increase of danger-safety ratings to CSP, *F*_(1, 18)_ = 5.33, *p* < 0.05, η^2^ = 0.23, while danger-safety ratings did not change for CSM, *F*_(1, 18)_ = 1.00, *p* = 0.33. A Wilcoxon signed rank test comparing the last CSP extinction trial with the first CSP reextinction trial confirmed that ratings shifted to higher scale-categories following the avoidance test, *Z* = −2.13, *p* < 0.05.

### Skin conductance

#### Fear conditioning

Figure 3C suggests successful differential SCR, which is confirmed by a 2 (Group) × 2 (CS, averaged over the two trials) RM-ANOVA, revealing a main effect of CS, *F*_(1, 18)_ = 16.76, *p* < 0.01, η^2^ = 0.48.

#### Avoidance conditioning

Figure 3C suggests differential SCR that decreases over trials, which is confirmed by a 2 (Group) × 2 (CS) × 8 (Trial) RM-ANOVA, revealing a main effect of CS, *F*_(1, 17)_ = 9.86, *p* < 0.01, η^2^ = 0.37, and a main effect of Trial, *F*_(7, 119)_ = 3.59, *p* < 0.01, η^2^ = 0.17, as well as a CS × Trial interaction, *F*_(7, 126)_ = 2.25, *p* < 0.05, η^2^ = 0.12.

#### Transition from avoidance conditioning to response prevention

Figure 3C suggests an increase of differential SCR upon removal of the avoidance button, which is confirmed by a 2 (Group) × 2 (CS) × 2 (Trial: last avoidance, first extinction) RM-ANOVA, revealing a main effect of CS, *F*_(1, 18)_ = 27.17, *p* < 0.001, η^2^ = 0.60, a main effect of Trial, *F*_(1, 18)_ = 22.60, *p* < 0.001, η^2^ = 0.56, and most importantly, a significant CS × Trial interaction, *F*_(1, 18)_ = 13.11, *p* < 0.01, η^2^ = 0.42.

#### Response prevention and extinction

Focusing on the first eight trials of extinction, Figure 2C suggests gradual extinction of differential SCR, which is confirmed by a 2 (Group) × 2 (CS) × 8 (Trial) RM-ANOVA, revealing a main effect of CS, *F*_(1, 18)_ = 20.71, *p* < 0.001, η^2^ = 0.54, a main effect of Trial, *F*_(7, 126)_ = 7.55, *p* < 0.001, η^2^ = 0.30, and most importantly a significant CS × Trial interaction, *F*_(7, 126)_ = 4.65, *p* < 0.001, η^2^ = 0.21. In order to compare the end-points of extinction between the groups, we compared the last four extinction trials in a 2 (Group) × 2 (CS) × 4 (Trial: ext5-8 in Group ShortExt, ext9-12 in Group LongExt), revealing a significant main effect of CS, *F*_(1, 18)_ = 4.91, *p* < 0.05, η^2^ = 0.22, with no significant Group × CS interaction, *F*_(1, 18)_ = 0.00, *p* = 0.96. This suggests incomplete extinction, equally across groups.

#### Avoidance test

Figure 3C suggests that the return of the avoidance button had no detectable impact on the level of differential SCR. We calculated the average SCR for each CS during the four avoidance test trials and compared this with the averaged last four extinction trials (ext5-8 in Group ShortExt, ext9-12 in Group LongExt). The resulting 2 (Group) × 2 (CS) × 2 (Phase: last extinction trials vs. avoidance test trials) revealed a significant main effect of CS, *F*_(1, 18)_ = 12.76, *p* < 0.01, η^2^ = 0.42, with no CS × Phase interaction, *F*_(1, 18)_ = 1.52, *p* = 0.23.

#### Reextinction test

Figure 3C suggests a general increase in SCR upon removal of the avoidance button, which is confirmed by a 2 (Group) × 2 (CS) × 2 (Trial: last avoidance test trial, first post-avoidance test trial) RM-ANOVA, revealing a main effect of CS, *F*_(1, 18)_ = 10.12, *p* < 0.001, η^2^ = 0.36, a main effect of Trial, *F*_(1, 18)_ = 6.73, *p* < 0.05, η^2^ = 0.27, but no significant CS × Trial interaction, *F*_(1, 18)_ = 0.19, *p* = 0.67. A 2 (Group) × 2 (CS) × 2 (Trial: last extinction trial, first post-avoidance test trial) RM-ANOVA revaeled a main effect of CS, *F*_(1, 18)_ = 10.18, *p* < 0.01, η^2^ = 0.36, but no CS × Trial interaction, *F*_(1, 18)_ = 0.75, *p* = 0.40. A 2 (Group) × 2 (CS) × 2 (Phase: averaged last four extinction trials, averaged post-avoidance test trials) RM-ANOVA, yielded similar results, a main effect of CS, *F*_(1, 18)_ = 8.02, *p* < 0.05, η^2^ = 0.31, but no CS × Phase interaction, *F*_(1, 18)_ = 0.44, *p* = 0.52.

### Correlations with questionnaire scores: experiments 1 and 2 combined

Experiment 1 and 2 are exactly identical up until the avoidance conditioning phase. In order to explore effects of anxiety-related personality, we collapsed the two data from the two experiments and calculated correlations between the rates of button-clicking during avoidance conditioning and individual questionnaire scores (see Table 1 for a summary of descriptive statistics). None of the questionnaire scores correlated with the proportion avoided CSP trials, STAI-T: *r* = 0.11, *p* = 0.51, IU: *r* = 0.26, *p* = 0.11, CBAS: *r* = −0.05, *p* = 0.77 (uncorrected *p*-values). However, STAI-T did correlate with the proportion avoided CSM trials, STAI-T: *r* = 0.35, *p* < 0.05, IU: *r* = 0.13, *p* = 0.41, CBAS: *r* = 0.22, *p* = 0.17 (uncorrected *p*-values). Interestingly, this correlation was no longer significant following RPE treatment, when avoidance was available again, *r* = −0.02, *p* = 0.90, and STAI-T correlated significantly with the decrease in proportion avoided CSM trials between the avoidance conditioning phase and the avoidance test phase, *r* = 0.46, *p* < 0.05 (Bonferroni-corrected across eight correlation tests), while there was no such correlation with CSP trials, *r* = −0.12, *p* = 0.46. These results suggest that, although it was not able to wipe out avoidance altogether, RPE treatment did attenuate rates of unnecessary avoidance in higher anxious participants.

**Table 1:** **Descriptive statistics of anxiety-related personality trait questionnaires**.

	**State-Trait Anxiety Inventory (STAI)**	**Intolerance of Uncertainty Scale (IUS)**	**Cognitive-Behavioral Avoidance Scale (CBAS)**
Mean	37.65	66.35	53.78
Standard Deviation	8.22	18.29	15.13
Range	23–60	27–107	31–83

### Comparing the effects of fear extinction across experiments 1 and 2

The only difference between Experiments 1 and 2 is the operationalization of response prevention (removal of the button in Experiment 1, instructions in Experiment 2).

This allowed us to examine differences in efficacy of these two response prevention treatments on persistence of avoidance. Figures 2A,3A suggest that instruction-based response prevention may have been generally more effective than actual removal of the button. This was confirmed by a 2 (Experiment) × 2 (Phase: avoidance conditioning, avoidance test) × 2 (CS, averaged per phase) ANOVA that revealed a significant Experiment × Phase interaction, *F*_(1, 38)_ = 7.13, *p* < 0.05, η^2^ = 0.16, with no triple interaction, *F*_(1, 38)_ = 0.18.

## Discussion

The results of Experiment 2 are strikingly similar to those of Experiment 1: Differential danger-safety ratings and SCR returned sharply when participants were suddenly told that the avoidance button was unavailable, followed by gradual extinction (RPE). Subsequent instructions of renewed availability of the avoidance button led to a return of avoidance rates, indicating limited effects of PRE on avoidance when the response prevention is lifted. Finally, instructions of renewed unavailability of the avoidance button triggered an increase of extinguished differential danger-safety ratings in the Short Extinction group. This effect was not observed in the Long Extinction group, which may suggest that longer extinction could prevent this avoidance-induced increase of threat appraisal. Together, these results suggest that RPE effects are not only disturbed by visual changes (button present/absent, Experiment 1), but also by changes in instructed beliefs about avoidance availability. Exposure treatments also rely on therapist-patient instructions to exclude avoidance behaviors during exposures (RPE). Hence, the current results could imply that pure RPE treatments have limited effects on avoidance rates in everyday contexts where the avoidance options are typically available.

Over the two experiments combined, individual trait anxiety (STAI-T) correlated with the proportion of avoided CSM trials during avoidance conditioning, while there was no significant correlation with CSP trials. This finding adds to the diagnostic validity of the current procedure, and calls for studying avoidance responding during safety cues, rather than danger cues, in pre-clinical research on anxiety (see also Lommen et al., 2010; van Meurs et al., 2014). Interestingly, RPE treatment did decrease the proportion avoided CSM trials from avoidance conditioning to avoidance testing, and the size of the decrease correlated with STAI-T. This positive outcome may indicate that although RPE treatment failed to substantially reduce avoidance during a conditioned danger cue, it has the power to reduce the maladaptive avoidance during safety cues that characterizes high anxious individuals. Finally, comparing the two experiments directly revealed that instruction-based response prevention was generally more effective in reducing persistent avoidance than removing the response button. This may suggest that the learning not to avoid may be more effective in the presence vs. absence of avoidance cues.

## General discussion

The current study was set up to investigate response prevention and extinction (RPE) in a human avoidance conditioning protocol, and to assess its effects on the rate of avoidance when the response prevention was subsequently lifted. In line with a recent rodent study (Bravo-Rivera et al., 2015), we found persistent avoidance following fear extinction. Avoidance consisted of mouse clicking a button on the computer screen that appeared during both a conditioned danger (CSP) and safety cue (CSM). During avoidance conditioning, (1) participants learned to perform the avoid action more during CSP compared to CSM trials, (2) levels of danger-safety ratings and skin conductance reactivity (SCR) decreased as participants learned to avoid effectively, and (3) individual levels of trait anxiety correlated positively with unnecessary avoidance actions during the conditioned safety cue (CSM). Subsequent response prevention by removal (Experiment 1) or instructed unavailability (Experiment 2) of the avoidance button triggered a sharp increase in danger ratings and SCR to the CSP, followed by gradual reduction (extinction). Reintroduction of avoidance availability triggered a strong return of differential avoidance responding (CSP vs. CSM), but less so in Experiment 2 where response prevention had been induced through instructions while the avoidance button was always present. Finally, differential danger-safety ratings and SCR remained low during avoidance testing, but danger-safety ratings increased again when the avoidance availability was subsequently removed. Together, these results show that RPE effects can be studied in a human avoidance protocol and that lifting the response prevention can renew avoidance behaviors and lead to renewed expectancy of harm. The current study sets the stage for more research on avoidance extinction in humans and on developing/screening techniques to enhance transfer of RPE effects across contexts of avoidance (un)availability.

Several mechanisms may have contributed to the return of avoidance following RPE. Since fear is a motivator of avoidance (Krypotos et al., 2015), the return of avoidance may stem from a recovery of extinguished fear. Indeed, fear extinction is known to be specific to the spatio-temporal context in which extinction learning occurred (Bouton, 2002); the current RPE results suggest that it may also remain specific to the “context” of avoidance unavailability. The return of avoidance availability, through reappearance of the button or through instructions, may have functioned as a context change that triggered a recovery of fear and therefore avoidance as well. We found some support for this hypothesis during the subsequent removal of avoidance availability, which triggered a significant return of differential danger-safety ratings. Although this test was formally in a context identical to RPE, the preceding contextual changes may have disturbed that extinction context. An alternative possibility is that avoidance availability signals both safety and threat, as the avoidance action can become associated with the feared event (threat) that it effectively prevents (safety). Support for the safety-signaling hypothesis comes from recent studies on the predictive effects of avoidance actions (Lovibond et al., 2008, 2013). Support for the threat-signaling hypothesis comes from a recent study where performing an avoidance action during a conditioned safety cue elicited increased threat appraisal (Engelhard et al., 2015). Hence, the mere return of avoidance availability may have increased threat appraisal and fear, and thereby triggered the return of avoidance.

To the extent that the current RPE protocol relates to exposure treatment, the results would imply that conducting exposures in an avoidance-free therapy context enhances fear extinction within that context, but may compromise its generalizability to everyday contexts that have routine avoidance availability. Indeed, the presence of avoidance cues during fear extinction decreased the persistence of avoidance in Experiment 2. Incorporating the immediateness of avoidance availability in treatment may enhance extinction generalization (1) by augmenting the similarity with everyday contexts, and (2) by targeting the threat-signaling properties of avoidance cues. The judicious use of so-called safety behaviors in treatment is an ongoing question in clinical exposure research, with mixed evidence and diverging opinions (Rachman et al., 2008). The current analysis adds to this literature by pointing to the potential influence of avoidance (un)availability on the generalization of fear extinction and avoidance extinction.

The most important limitation of the current study is that fear extinction was not complete in the two experiments, even after as much as twelve extinction trials (compared to two Pavlovian conditioning trials). The level of differential danger-safety ratings and SCR did decrease significantly over extinction trials, but remained significant over the last four trials of extinction. This delayed extinction effect could result from the instructed fear procedure (participants were informed beforehand which CS would be followed by shock), if instruction-based learning is more difficult to correct by experienced-based learning. Alternatively, the avoidance conditioning trials may have strengthened the underlying CS-US association, leading to slower extinction. The latter hypothesis could be investigated by dropping the contingency instructions and by manipulating the amount of avoidance conditioning trials prior to extinction. Irrespective of the exact mechanism leading to slower extinction, it is possible that the residual fear levels were responsible for the subsequent return of avoidance. This would show that, at least in the case of low-cost avoidance behavior, minimal levels of fear are sufficient to trigger a return of avoidance behavior when the opportunity arrives. In that case, preventing return of low-cost avoidance behaviors would require a complete elimination of fear reactions during treatment, as well as a complete prevention of return of fear.

A second limitation is that the avoidance response carried no cost. This is different from clinical avoidance behaviors, which are often very costly as they prevent the patient from engaging in other desired activities. Future research should investigate the influence of response costs on the return of avoidance after RPE. Nevertheless, as we suggested in the Introduction section, a patient may use many different avoidance behaviors, some of which are costly and some of which are not. The costly avoidance behaviors will be salient and probably be part of the primary complaints of the patient and an explicit target of treatment. Low-cost avoidance behaviors, on the other hand, may be more difficult to detect and therefore more difficult to treat. And, as the current study suggest, they may be especially prone to persist after fear extinction.

To conclude, fear extinction is generally viewed as an experimental model of exposure treatment, but the critical component of response prevention is often lacking. The current study established a human avoidance protocol in order to study fear extinction following a history of avoidance, and examined the effects of a return of avoidance availability on the return of avoidance responding. The results suggest that avoidance responding returns easily following fear extinction. This calls for more extinction research focusing on rates of avoidance behaviors in addition to levels of fear reactions. Arguably, incorporating avoidance into the learning history as well as in the extinction test situation may enhance the external validity of the fear extinction model and improve its translational value to the clinical setting.

## Author contributions

BV developed the research questions. BV and EI designed the experimental procedures and analyzed the data. BV wrote the manuscript, commented by EI, BV. EI approved of the final version and agreed to be accountable for all aspects of the work.

## Funding

This research was supported by a Marie Curie International Outgoing Fellowship within the 7th European Community Framework Programme (PIOF-GA-2013-627743) and by a Center for Excellence grant from the University of Leuven—KU Leuven (PF/10/005).

### Conflict of interest statement

The authors declare that the research was conducted in the absence of any commercial or financial relationships that could be construed as a potential conflict of interest.
