# Corrigendum: Low-Cost Avoidance Behaviors are Resistant to Fear Extinction in Humans

**DOI:** 10.3389/fnbeh.2021.656847

**Published:** 2021-02-25

**Authors:** Bram Vervliet, Ellen Indekeu

**Affiliations:** ^1^Department of Psychiatry, Massachusetts General Hospital and Harvard Medical School, Boston, MA, United States; ^2^Center for Excellence on Generalization in Health and Psychopathology, University of Leuven—KU Leuven, Leuven, Belgium; ^3^Department of Psychology, University of Leuven—KU Leuven, Leuven, Belgium

**Keywords:** fear, extinction, avoidance, response prevention, exposure

In the original article, there was a mistake in Figure 3 as published. Panel C of Figure 3 presented the skin conductance results of Experiment 1, instead of Experiment 2. The corrected Figure 3 appears below.

**Figure 3:**
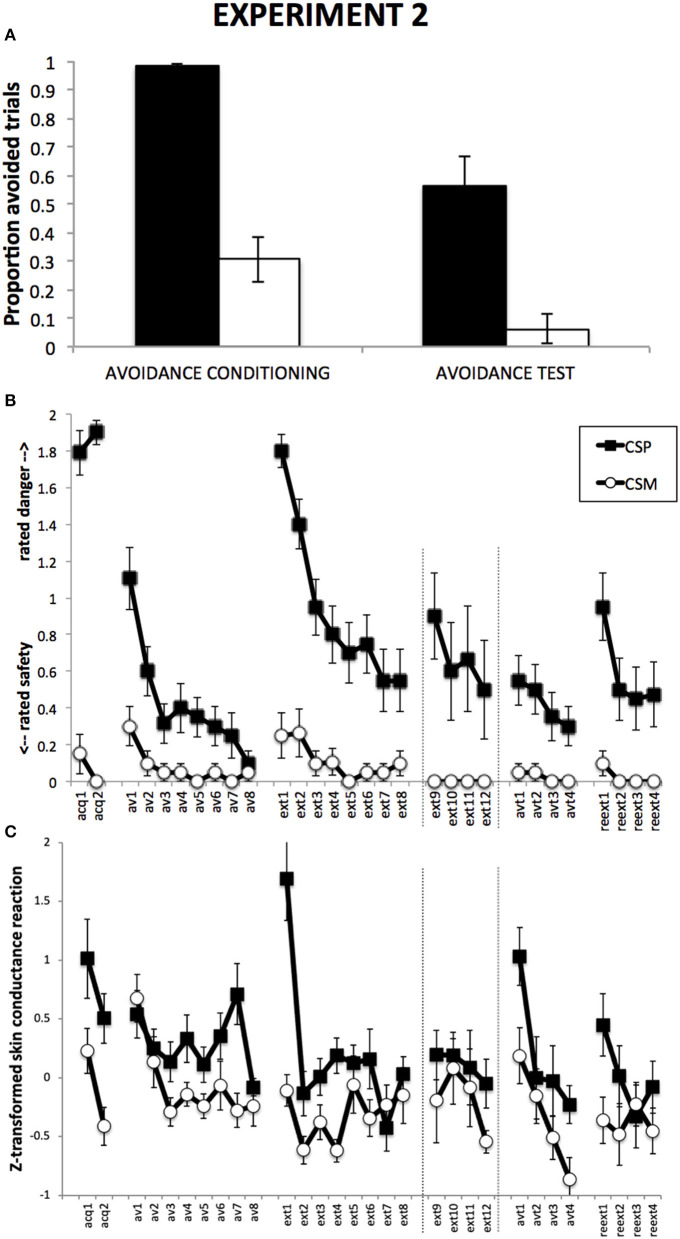
Results from Experiment 2. **(A)**: Proportions of CSP and CSM trials during which the avoidance button was clicked, for avoidance conditioning and avoidance test phase separately. **(B)**: Mean shock-expectancy ratings during CSP and CSM (0 = “safe,” 1 = “uncertain,” 2 = “danger”), for all trials of Pavlovian conditioning (acq1-2), avoidance conditioning (av1-8), response prevention and extinction (ext1-8) with extension for Group ExtLong between the dashed lines (ext9-12), avoidance test (avt1-4), and reextinction (reext1-4). **(C)**: Z-transformed skin conductance reactions during CSP and CSM during all trials (cf. **B**). Error bars represent standard errors of the means.

The authors apologize for this error and state that this does not change the scientific conclusions of the article in any way. The original article has been updated.

